# Mobile consulting (mConsulting) and its potential for providing access to quality healthcare for populations living in low-resource settings of low- and middle-income countries

**DOI:** 10.1177/2055207620919594

**Published:** 2020-04-20

**Authors:** Frances Griffiths, Jocelyn Anstey Watkins, Caroline Huxley, Bronwyn Harris, Jonathan Cave, Senga Pemba, Beatrice Chipwaza, Richard Lilford, Motunrayo Ajisola, Theodoros N. Arvanitis, Pauline Bakibinga, Muntasir Billah, Nazratun Choudhury, David Davies, Olufunke Fayehun, Caroline Kabaria, Romaina Iqbal, Akinyinka Omigbodun, Eme Owoaje, Omar Rahman, Jo Sartori, Saleem Sayani, Komal Tabani, Rita Yusuf, Jackie Sturt

**Affiliations:** 1 Warwick Medical School, University of Warwick, UK; 2 Centre for Health Policy, University of the Witwatersrand, South Africa; 3 Department of Economics, University of Warwick, UK; 4 St Francis University College of Health and Allied Sciences, Tanzania; 5 University of Ibadan, Nigeria; 6 Institute of Digital Healthcare, WMG, University of Warwick, UK; 7 African Population and Health Research Centre, Kenya; 8 Independent University Bangladesh, Bangladesh; 9 Aga Khan University, Pakistan; 10 The Florence Nightingale Faculty of Nursing, Midwifery & Palliative Care, King’s College London, UK

**Keywords:** mHealth, mConsulting, mobile consulting, remote consultation, healthcare, low-and middle-income countries, slums, rural areas

## Abstract

**Objective:**

The poorest populations of the world lack access to quality healthcare. We defined the key components of consulting via mobile technology (mConsulting), explored whether mConsulting can fill gaps in access to quality healthcare for poor and spatially marginalised populations (specifically rural and slum populations) of low- and middle-income countries, and considered the implications of its take-up.

**Methods:**

We utilised realist methodology. First, we undertook a scoping review of mobile health literature and searched for examples of mConsulting. Second, we formed our programme theories and identified potential benefits and hazards for deployment of mConsulting for poor and spatially marginalised populations. Finally, we tested our programme theories against existing frameworks and identified published evidence on how and why these benefits/hazards are likely to accrue.

**Results:**

We identified the components of mConsulting, including their characteristics and range. We discuss the implications of mConsulting for poor and spatially marginalised populations in terms of competent care, user experience, cost, workforce, technology, and the wider health system.

**Conclusions:**

For the many dimensions of mConsulting, how it is structured and deployed will make a difference to the benefits and hazards of its use. There is a lack of evidence of the impact of mConsulting in populations that are poor and spatially marginalised, as most research on mConsulting has been undertaken where quality healthcare exists. We suggest that mConsulting could improve access to quality healthcare for these populations and, with attention to how it is deployed, potential hazards for the populations and wider health system could be mitigated.

## Introduction

### The problem: lack of access to high-quality healthcare for the poorest populations in low- and middle-income countries

It is a United Nations sustainable development goal (SDG) to achieve universal health coverage and access to quality healthcare for all.^1^ With the poorest populations of the world still lacking access to quality healthcare provision,^2^ it is ‘time for a revolution’.^3^

In many low- and middle-income countries (LMICs), it is the quality of the care provided, as much as the ability to access the care, that is a key problem.^3^ Marginalised populations have the least access to high-quality services; populations including those living in informal settlements and migrant populations, people with stigmatised conditions (such as HIV/AIDS, mental disorders and substance abuse), those who experience power inequalities (such as women and people with disabilities), those with little education or income, and people living in rural areas.^3^ Furthermore, perceptions and experiences of low-quality care may prevent people from seeking care when it is needed.^3^

Nearly one billion people live in slums.^4^ In sub-Saharan Africa, the proportion of urban residents living in a slum is particularly high (56%).^4^ In slums, there are often high rates of population turnover, high crime rates and exposure to violence, which often result in death, injury, or mental illness.^5^ Slum dwellers may be physically close to healthcare services, but the quality of care is likely to be low. For rural populations, access to healthcare is a continuing problem, with shortages of healthcare providers and weak infrastructure, including transport routes. This has an impact on access to services, particularly for impoverished rural populations.^6^

This paper considers poor communities who are marginalised spatially by their physical remoteness or by being slum dwellers.

Our proposition: mConsulting has a key role in improving access to quality healthcare for poor and spatially marginalised populations in low- and middle-income countries

We suggest mConsulting can contribute to ‘the revolution’, by improving access^7^ to quality healthcare for poor and spatially marginalised populations. We argue that, with attention to how it is deployed, potential hazards for the populations and wider health system^8^ can be mitigated. There is no standardised definition for the concept of mConsulting. We start to unpack what it means in Box 1 and throughout this paper.

Box 1 What is mConsulting?

Our definition of mConsulting is when a person with a perceived health need consults a healthcare provider using mobile communication technology, or a provider contacts their patient. For example, a woman accesses an interactive website for advice about family planning, or a man sends a text message (SMS) to a clinic physician to request an anti-malarial prescription or a nurse contacts a patient with their test results. While mobile technology would usually be the means of communication, we do include consultations using non-mobile technology (e.g. a computer in a community centre or a shared fixed telephone line in a remote rural village), where the access is to services that are usually considered mConsulting. We also include the scenario where the person with a health need asks an intermediary, such as a relative or community health-worker, to assist them with mConsulting. We propose this expanded definition of mConsulting in recognition that digital communication technology is not yet ubiquitous and, for some populations, these variations may persist because of a lack of resources or logistics. We are not including situations where a healthcare provider assesses the patient themselves, then separately seeks advice from someone with more expertise.

Globally there has been an unprecedented uptake of digital communication technology. This has been facilitated by technological advances, network coverage, and relatively affordable digital services. Mobile phone ownership is estimated at 85% across all LMICs,^9^ with 75–90% of Africans^10^ and two-thirds of Asians estimated to own mobile phones. There are, however, differences in ownership levels between and within countries, with those from the poorest countries (such as Mozambique) and most marginalised groups (women, the less educated, rural and poorer sectors of the population) less likely to own a mobile phone.^9–11^ Women also tend to use a narrower range of mobile services and spend less on service usage than men.^9^ However, in the last 3 years, the gender gap has narrowed and 80% of women in LMICs are now estimated to own mobile phones.^9^ There is growing commercial and policy interest in the transformative potential of digital technology to reduce gender, geographical, institutional, and financial barriers to healthcare, to strengthen health systems, improve health outcomes and enable countries to move towards universal health coverage.^12–18^

There is some evidence that use of mConsulting, mostly within the private health sector, is starting to emerge in low-resource communities.^16,19,20^ However, evidence of the contribution of mConsulting to population health and health system strengthening is limited. The role of the private health sector in LMICs is under-researched and highly complex. Provider types range from those operating in the ‘low-quality, underqualified sector that serves poor people in many countries’, through not-for-profit organisations and small-to-medium enterprises, to the ‘corporate commercial hospital sector’^21^ (p. 622). The telecommunication industry is also an important actor, along with companies set up specifically to provide mConsulting (e.g. Babylon,^22^ Babyl^23^ and Ada Health^24^). Private-sector provision has the potential to facilitate access to required care in low-resource communities, particularly those that are underserved by the state, but this will depend on the quality, purpose, affordability and acceptability of the care provided, and how private provision is situated in the health system as a whole.^21^

### Mobile digital communication technology for health in LMICs

Evidence from LMICs indicates that use of digital communication technology for health can improve management of chronic and non-communicable diseases,^13,25,26^ increase patient utilisation of maternal and neonatal services,^27^ bring about positive change to adolescent sexual behaviour,^28^ and increase access to previously unavailable services.^29^ The use of text message reminders has resulted in increased vaccination coverage among rural hard-to-reach communities and urban street-dwelling communities.^30^ However, despite extensive research literature, recent reviews indicate there is little empirical evidence of mHealth service availability, or use and perceptions amongst poor and marginalised communities. Who is using what services and why?^12,16,26,31^ World Health Organization (WHO) recommendations on digital interventions for health system strengthening^18^ have little to say about the use of mConsulting in such communities, given that the recommendations are largely based on services in high- and upper-**middle** income countries, where mConsulting is supplementary to existing service. The WHO evidence review team found no evidence of resource use for mConsulting in the effectiveness studies they included, instead basing their information on programme documents and discussions with people implementing mConsulting services.^18^ They concluded that mConsulting is not a good alternative, given the magnitude of resources required; but this appears to be in relation to mConsulting services that are independent of any existing services. The lack of policy attention given to the use of mobile communication technology to improve health in poor populations^12,16,32^ is starting to change, with the publication of the WHO Draft Global Strategy on Digital Health 2020–2024.^33^ Provision of mConsulting via the private sector could, arguably, overcome certain constraints on a country’s health system.^34^

The use of mobile communication technology for health is currently hampered in LMIC settings by uneven/poor network connectivity, rapid technological change, low (technological) literacy levels amongst users, and limited awareness of available services.^12,18,29,35^ There are also concerns about obtaining informed consent and data security.^18^ However, patients and healthcare providers have found mConsulting acceptable. Nonetheless, some healthcare providers have expressed concerns that the quality of care may be lower than when face-to-face,^18^ however this was expressed where quality face-to-face care existed.

### The study

Our aim was to

- describe the concept of mConsulting, including its changing nature and boundaries, what it looks like in reality and how reality shapes it;
- explore the value of mConsulting and the potential benefits and hazards when deployed for poor and spatially marginalised populations.

Our question was: To what extent can mConsulting fill a gap in access to quality healthcare for poor and spatially marginalised populations of LMICs, and what are the implications of its take-up for the target population and for health systems?

## Methods

In order to explore the contribution and impact of mConsulting in LMICs, we adopted a realist review (or realist synthesis) approach^36,37^ to answer the question ‘what works for whom, under what circumstances, how and why?’,^37^ by synthesising heterogenous evidence from a range of diverse contexts.^38^ We took a grounded approach, drawing on published evidence and the expertise of an international team of researchers working in Bangladesh, Kenya, Nigeria, Pakistan, Tanzania and the UK, with experience of research and healthcare provision in slum and rural communities, and from a range of disciplinary backgrounds (including public health, medical sociology, health science, behaviour change, health service research, digital technology innovation, behavioural economics and clinical science). We first undertook a scoping review of the mHealth literature and searched grey literature on the Internet for examples of mConsulting and its evaluation (led by JAW). Informed by this, we held a workshop, involving all co-authors (except JAW and CH), to form our programme theories^36^ of mConsulting and the potential benefits and hazards for its deployment for poor and spatially marginalised populations in LMICs. We considered both intended and unintended consequences,^39^ including its impact on health, how health is perceived and managed, and implications for the population and the health service, including health economics. We considered why mConsulting may bring advantages or risks and what might enhance or diminish these risks. We also considered what *could* arise and the *specific ways* this might affect patients, healthcare providers, and service providers. We then tested our programme theories against existing frameworks^3,40,41^ and identified published literature to provide evidence on how and why these benefits/hazards are likely to accrue.

## Results

### mConsulting as a complex adaptive system – a conceptual framework

Where mobile communication technologies have been introduced, such as in banking and shopping, systems have changed: the supply, demand and mechanisms by which users and providers find each other, and the ways in which they are monitored and followed up or not. The introduction of mConsulting potentially affects the whole system; it is not an isolated service innovation.^21^ To understand mConsulting, we therefore need to understand health and technological systems as complex adaptive systems^42^: dynamic, self-regulating, non-linear, context-bound and not always predictable.^43^ Through the use of information technology, events are not ‘bounded by conventional notions of time/space including who can participate, what happens and where it can happen’^44^ (p. 62), but nevertheless interact with the physical reality of people’s lives, their motivations and behaviours.^44^ mConsulting may feel different to face-to-face consulting and the implications of this are not yet known.^45^ High-quality healthcare adopts innovation and adapts to societal change.^3^ We therefore argue that in the context of poor and spatially marginalised communities, mConsulting has the potential to precipitate non-linear change and feedback that could result in significant change to the community, the health system and the policy environment. These changes could contribute to healthcare that is ‘for people and is equitable, resilient and efficient’,^3^ although, in every complex adaptive system, there are always unintended consequences and potential hazards, as well as anticipated benefits.^42^

**Figure 1.**
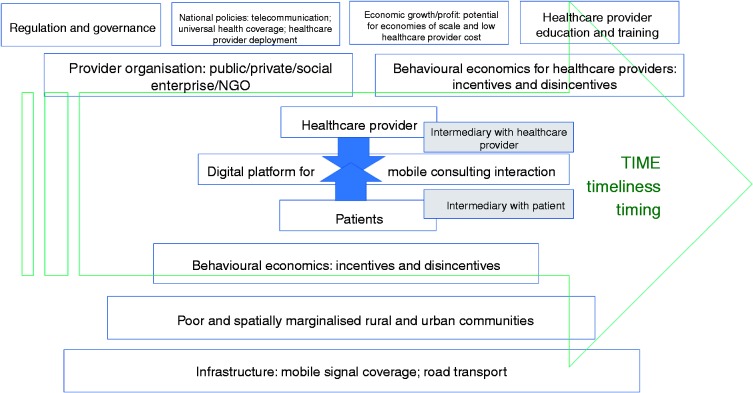
mConsulting as a two-sided complex adaptive system involving healthcare providers and patients.

Healthcare can be considered a two-sided network, with providers and patients connected across an interaction platform,^46^ which, in the context of mConsulting, is a digital communication platform (see Figure 1). Time is an important dimension for mConsulting. Digital communication is changing our understanding of time.^47^ Furthermore, digital technology itself is rapidly changing, as are associated behaviours, systems, policies and expectations.

### Characterising mConsulting as a complex adaptive system

#### The digital platform for mConsulting

Platforms range from a mobile phone call made by a patient to their healthcare provider (e.g. Aponjon, a mobile consulting service for maternal, neonatal and infant healthcare in Bangladesh^48^), to major commercial companies, communicating with their own platform, such as Babyl,^23^ which currently operates in Rwanda (as well as in the UK as Babylon^22^). Individuals might use their own phone or one borrowed from a friend, or they might go to their local community centre to use a computer. Healthcare providers might use their own hardware or have it provided by an mConsulting service.

#### The patient

The patient engaging across the platform recognises themselves as having a health need^49^ and this can be any type of health need. They can be located anywhere where they can, at the very least, walk to a place with an available mobile phone signal; if they can afford it, they may have access to 4G, which is possible in some urban slums. They can be of any age, once capable of managing the technology and the consultation. Gender has relevance where it influences access to healthcare, for example, a woman needing permission from her husband to seek healthcare or requiring a female healthcare provider, or a man who works away from home, for example, a truck driver. Both men and women work long hours in their employment. The patient needs sufficient health and digital literacy to engage with mConsulting, or they need an intermediary, such as a family member or community healthcare provider, to help them with the consultation. Uncompensated and untreated eyesight problems can prevent use of text-based communication, and poor dexterity can make use of communication technology difficult.^50^ For any consultation, the languages spoken are important, particularly as the patient and healthcare provider may be located at considerable distance from each other.

#### The healthcare provider

The healthcare provider may be of any type. The patient considers them as someone who might have expertise relevant to their health need. Location, gender, languages spoken, and the presence of an intermediary are also relevant for the healthcare provider. The healthcare provider may have experience in any of the current health sectors, including primary care, specialist care, and traditional medicine. The healthcare provider might have formal qualifications as a doctor, nurse, Unani or Ayurvedic practitioner, pharmacist, community healthcare provider or they may have experienced-based or on-the-job training. Healthcare providers such as doctors and nurses are required to be registered, but registration of traditional healers varies by country.^51^ The healthcare provider could be a chatbot, driven by algorithms (rule-based or machine learning algorithms). Healthcare providers may limit their services to those that can be delivered virtually (e.g. by offering virtual triaging to support patient decision-making for further health actions^24^) or they may link their services to providers who offer face-to-face care (e.g. Babyl Rwanda,^23^ DoctHERS Pakistan^52^).

#### The content of the consultation

Adapting the definitions in the WHO Classification of Digital Health Interventions,^15^ which focuses on content, the following could be undertaken through mConsulting:

- Consultations between remote client and healthcare provider;
- Remote monitoring of client health by healthcare provider;
- Transmission of medical data to healthcare provider by client;
- Transmission of diagnostic result to client.

On the boundary of mConsulting is ‘client look-up of health information’,^15^ where there is a system for tailoring the information offered to what the user asks. With the growth of algorithm-driven systems, this type of mConsulting may become more common in the future. The content may relate to any type of health need, including urgent/non-urgent, first episode or ongoing health issue, and preventive healthcare.

Many diagnostic tests can be arranged remotely, where the relevant kit is available, for example, blood pressure and pulse, finger-prick blood tests, and swabs. Where physical examination and diagnostic tests (such as X-ray, endoscopy and biopsy) are required, further arrangements are needed to ensure the patient has access to these.

#### Relationship of the patient and healthcare provider

The patient may have already seen the healthcare provider face-to-face, or others working in the same service provider context, such as in a clinic. They may know the healthcare provider as a neighbour or friend. Alternatively, the patient may contact a healthcare provider where there is no pre-existing relationship, following a recommendation, prompted by advertising, or from searching for a provider.

#### Timing and timeliness of interaction

Mobile communication gives both patient and healthcare provider flexibility about when and where they interact. There is potential for both of them to fit mConsulting around their other commitments. For example, healthcare providers can consult in the evening, when their children are asleep and their patients are home from work. However, some healthcare providers may want to confine mConsulting to their current work patterns.

mConsulting has the potential to be timely, in relation to the patient’s health need. This might be for urgent issues such as injury. For people living with long-term conditions, mConsulting can enable self-management through provision of timely access to their healthcare provider for consultation.^53^

As with face-to-face consultations, it will take patients time to access mConsultations: contacting the service, making a booking, waiting in a queue. The nature of this experience will depend on how the mConsulting service is configured. There is the potential for patients to continue with their day-to-day activities while awaiting mConsultation, however, this is likely to depend on their context, including their ability to find a private space for consulting.

In Table 1 we summarise the key characteristics of mConsulting.

**Table 1.** The components of mConsulting, their characteristics and range.

Component of healthcare network	Characteristics relevant to mConsulting and their range
Digital communication platform	
Timing of interaction.	Synchronous/asynchronous.
Form of communication.	Audio/audio-visual/text/photo.
Specificity of platform.	Generic/provider-specific.
Hardware for digital access.	Phone/tablet/computer.
Ownership of hardware.	Owned/borrowed/shared in household/communal/provided by intermediary/provided by mConsulting or other service.
Patient engaging across platform.	
Health need.	Patient recognises they have a need for healthcare – this can be any need.
Location.	Anywhere with mobile phone signal/cable, Wi-Fi or Internet access.
Personal characteristics.	Any age with sufficient health and digital literacy; gender where relevant, e.g. culture, employment/languages spoken/eyesight/dexterity.
Intermediary present with patient.	Layperson, healthcare provider or interpreter assisting patient during mConsultation.
Healthcare provider engaging across platform	
Type of healthcare provider.	Doctor/nurse/traditional healer/pharmacist/community health-worker/chatbot/and other healthcare providers (such as physiotherapists, midwives etc.).
Location.	Anywhere with mobile phone signal/cable, Wi-Fi or 4G Internet access.
Personal characteristics.	Gender where culturally relevant/languages spoken.
Intermediary present with healthcare provider.	Family member who has gone to ask for advice but finds more information is needed/interpreter/another healthcare provider providing expertise.
Qualifications/experience.	Formally recognised training/experienced-based training/no training.
Registration/regulation.	Required/not required.
Content of communication across platform	
Consultations between remote client and healthcare provider.	New/existing health condition, urgent/non-urgent.
Remote monitoring of client health by provider.	Recovery from acute illness/monitoring of long-term condition.
Transmission of medical data to healthcare provider by client.	New/existing health condition, urgent/non-urgent.
Transmit diagnostics result to clients.	New/existing health condition, urgent/non-urgent.
Client look-up of health information where there is a system for tailoring the information offered to what the user asks.	New/existing health condition, urgent/non-urgent.
Patient – healthcare provider relationship	
Existing relationship.	With individual healthcare provider or with service provider; formal or informal.
No pre-existing relationship.	Recommended by people in patient’s social network or other healthcare providers/advertisements/patient-initiated Internet search.
Timing and timeliness of patient – healthcare provider interaction	
Flexibility.	Limited to standard work hours and appointment times/ 24/7.
Timeliness.	Wait for appointment and in virtual waiting room somewhat like face-to-face consultation/access when patient perceives need for advice that makes a difference to self-management or other health decisions.
Time/disturbance to day-to-day activity to access mConsulting.	Varies according to configuration of service and patient context, including privacy.

### mConsulting service providers

Service providers include telecommunications companies, where mConsulting is a specialist area of provision, companies set up specifically for provision of mConsulting services, social enterprises and NGOs (e.g. Babyl in Rwanda,^23^ iafya in Kenya,^54^ and the 104 health helpline in Odisha, India^55^). mConsulting can also be provided by healthcare providers as an addition to their face-to-face services. Examples include telephone consultation with a doctor working in the private or public sector, a telephone call to a pharmacy for advice on medication, consultation with a traditional healer by phone or Skype, an email exchange with a physiotherapist or social worker, or an exchange of text messages with a nurse or community health-worker.

### Potential benefits and hazards of deploying mConsulting for poor and spatially marginalised populations

In Table 2, we present data from our workshop and published evidence of the potential benefits and hazards of the deployment of mConsulting for poor and spatially marginalised populations.

**Table 2.** Benefits and hazards of mConsulting for poor and spatially marginalised populations.

Component of healthcare^3^	Benefits of mConsulting	Hazards of mConsulting
Competent care	Contact with healthcare provider more available in terms of time and place for patient.Advice potentially available immediately or when timely for the patient so potential for earlier presentation of new symptoms or concerns leading to improved health outcome.^56,57^Patient education and self-learning/care may be enabled especially if the consultation is timely.^53^Medium of communication enables sharing of information.^53^ mConsulting platform may include prompts for people to look at/look up information for themselves. Consultations can be overtly or covertly recorded for patients to review later^58–60^ or for both healthcare and service providers to review quality. ^41,61,62^ Covert recordings can result in, or are a result of, lack of trust between provider and patient.^58,59^Where mConsulting is between patient and healthcare provider remote from each other patients may still hold expectations for medicinal solutions (albeit medicines not requiring a prescription from a healthcare provider).^48^Potentially lower barriers to follow-up and monitoring (e.g. no transport costs and travel time) so it is more likely to happen.Ability to bypass existing provision perceived as inadequate (e.g. healthcare providers’ unpleasant behaviours, lack of urgency, unnecessary delays and unauthorised fees).^63,64^ This may be particularly important for subgroups who are further marginalised within the poor and spatially marginalised population (e.g. sex workers).^65^ However, traditional power structures within the community or households may inhibit use of digital communication.^66^ The need to seek permission to access healthcare from family members is often linked to financial implications,^67^ so may not be mitigated by mConsulting.Healthcare provider/service provider may have access to online evidence-based guidance as already digitally connected or system built based on evidence.^68^Counselling can be provided effectively, without need for travel to face-to-face consultation.^69^Evaluation of consultations via videoconference suggests that when clinical, technical and practical preconditions were met, virtual consultations appeared to be safe; however, both parties sometimes needed to make explicit things that typically remained implicit in a traditional encounter.^41^	There are potential risks to patient safety from mConsulting as the healthcare provider may miss subtle clinical features that are easier to detect in person/on examination.Physical examination and complex diagnostics such as X-ray need to be arranged separately, which may be difficult for poor and spatially marginalised populations.Healthcare providers may be reluctant to assess certain types of health problem (e.g. musculoskeletal problems) via mConsulting even where videoconferencing available.^70^Treatments may be suggested that are not available locally.The healthcare provider faces the challenge of how to complete the consultation.^71^ With mConsulting the healthcare provider might tend to suggest trying a treatment as an easy option rather than getting the person to attend for a physical examination or other diagnostics.The healthcare provider may not be linked to local referral systems for specialist care.Digital communication might increase accessibility for patients to healthcare providers in their social networks for informal advice^72^ but with a risk of incomplete assessment.The healthcare provider may be unaware of other healthcare provision in locality of patient, how they can be used by the patient for accessing diagnostics and treatment and the most appropriate use of resources.^73^Patients may find it easier to present expected symptoms to gain what they want e.g. antibiotics.^74,75^
User experience	Communication using videoconferencing is, usually, as good as face-to-face, including for mental health.^76^Some patients with communication problems are helped by use of digital communication^18^:– Interpreters can be included in three-way communication.– Patients can directly access providers who speak their language.^73^– Potential choice of communication medium (text/voice/video).Advantage of anonymity allows patients to ask questions they might not otherwise ask.^64,77^Advantage of anonymity for patients wishing to discuss stigmatised conditions or sensitive topics (e.g. mental health, sexually transmitted diseases, sexual health, etc.). mConsulting means they avoid both being seen going to clinics^77^ and discussing their problem face-to-face.^77^ Providers can potentially ensure short waiting times for patients. There is potential for increased trust between patient and healthcare provider with use of mConsulting where a relationship already exists^53^ and where it does not, when anonymity encourages rapid disclosure.^78^Patients feel more comfortable consulting in their own home.^79^ Patients can develop a sense of entitlement which can benefit patients who have experienced being dismissed by face-to-face services.^80^Where patients have experienced overstretched face-to-face services and where their ability to assess the appropriateness of seeing healthcare providers has been questioned, mConsulting can have a role in empowering patients.^81^ Quality advice can be provided in emergency situations (e.g. advice given on how to apply a tourniquet for a snake bite, or telling patients where to go for treatment or when an ambulance will arrive).	Some patients with communication problems may be excluded or need an intermediary to assist due to:– Hearing impairment^82^– Poor dexterity^50^– Lack of digital literacy^82^– Poor literacy.^83,84^The healthcare provider may be less likely to pick up that they are having problems with communication.Difficulty in sensitively conveying bad news about their health to a patient (face-to-face preferred).^85,86^ Virtual consultations tend to be more doctor-centred compared with face-to-face consultations, which are more patient-centred.^87^ Their use may therefore reduce the patient-centredness of healthcare delivery.Patients/healthcare providers may not be certain that the person being consulted/consulting is who they say they are.Patients may not have access to a private space for the consultation,^88^ particularly in poor households and the close living of slums.The patient may be unable to judge whether the healthcare provider is paying sufficient attention to privacy/confidentiality at their end of the consultation (e.g. pharmacist in their shop).Patients can develop a sense of entitlement that can result in unnecessary use of the service.^80^Ease of referral of patients from one healthcare provider to another may increase referral rates (e.g. pharmacist referring to a doctor). Patients perceive that such referrals facilitate quicker, better-quality or discounted treatment.^67^Patients can be concerned that a healthcare provider consulting remotely with them may not take sufficient account of their living environment and culture when planning health condition management.^89^
Cost	Advice may be more affordable than face-to-face consultation.Saving on transport costs for patients^90^/providers.Consultations are usually shorter when people consult by phone/video (as they dispense with the niceties and are more transactional),^41^ potentially reducing consultation time and associated opportunity costs for both patients and providers.	Provision of advice may be linked to purchasing other products,^91^ drawing people into additional costs or other disbenefits.Need to have at least mobile phone and airtime, so certain sectors of the population may be excluded.Women, particularly women living in remote or rural areas, may have less access to mConsulting services because they are less likely to own mobile phones, use as many services or spend as much on mobile services as men.^9,11^
Workforce	Flexible deployment of healthcare providers benefits the health system (more healthcare providers available) and healthcare provider quality of life (e.g. working from home while children are asleep).Healthcare providers can provide care for spatially marginalised populations without needing to live local to the population.Healthcare providers do not need to travel to insecure or remote sites, which can solve the problem of providing healthcare to these populations.	Healthcare providers may experience increased workload^53^ and leave or retire early or feel unable to manage the change in practice to mConsulting.Healthcare providers may leave face-to-face services to work for mConsulting leaving a gap in face-to face services.
Technology	When people are already using technology as part of a consultation, it might be easier for them (as both patients and providers) to extend their use of technology further, e.g. to implement other forms of technological support (reminders, searches on health, etc.) or, beyond the individual relationship, to build in new technology, as part of the consultation or beyond (sensory devices, bringing in interpreters or intermediaries, using monitoring e.g. glucometer).The technology potentially allows patients to record their consultations so they can review them later for information, to check accuracy or to share with other people.^58^	Network coverage may be poor in rural locations.^92^Connection may be poor or breakdown, disrupting the consultation.Requirements of the mConsulting platform may be much greater than is common among poor populations.LMICs, and low-resource settings within national borders, are not always viewed as commercially viable settings for high-tech interventions and adaptations (they are not seen as cost-effective), e.g. modular phones, medical diagnostic sensors and so forth could be adapted beyond a smart phone but have not been.
Wider health system	Reduced pressure on overstretched clinics by removing unnecessary face-to-face appointments.^93^	The availability of a (more immediate) online/mobile interaction changes what symptoms people consider important enough to warrant seeking healthcare, compared with a (longer-awaited) face-to-face interaction, resulting in overuse of mConsulting services.The medium may start to facilitate a certain way of relating between patient and healthcare provider (e.g. patients may start to rely on not having to present in person) or the patient may expect a certain response from the provider (e.g. prescribing antibiotics).Where mConsulting is considered sufficient for remote and insecure locations, provision of face-to-face services and diagnostics could be withdrawn/not resourced.Unregulated services may dominate the market initially.Less regulated healthcare providers may take on mConsulting earlier than others.

Note: LMICs = low- and middle-income countries.

For many dimensions of mConsulting, how it is structured and deployed will make a difference to the benefits and hazards. The business model of the mConsulting provider is a key factor, for example, whether the service is affordable and for whom, how it is linked to other services/products, who provides the care, and the level of monitoring of care standards. Also important is the degree to which mConsulting services take account of the characteristics of the population they seek to serve, including cultural norms, literacy levels and language. mConsulting may provide continuity of care for patients where a healthcare provider or team has access to previously recorded patient information. This may be particularly important for transient populations, including those of slum communities.^94^ mConsulting, linked to digital transfer of monetary fees, might have the effect of standardising fees and reducing requests for ‘unauthorised’ fees. However, easy access to unregulated mConsulting may result in individuals receiving advice that is of poor quality, inappropriate or unnecessary. Informal, unstructured mConsulting can lead to ethical problems, including unclear professional boundaries, and uncertainty about duty of care outside of working hours.^18,95^ The provision (or not) of healthcare provider training in mConsulting and taking advantage (or not) of the opportunity to review consultations for quality and provide feedback, and to facilitate access to information and expertise will again make a difference to the benefits and hazards. If mConsulting is more affordable, this might lead to increased use amongst the poorest populations and, therefore, increase their exposure to the hazards of mConsulting. mConsulting could also create demand for a service that is not easily available to a community, for example, a diagnostic laboratory. This could frustrate community expectations or, equally, lead to initiation of such a service. Involvement of citizens from the community to be served in shaping the design and delivery^96^ of mConsulting, can minimise the hazards and maximise benefits.

The impact of mConsulting will also be influenced by the population and context of implementation. For example, in countries where the public system of healthcare provision requires upfront payment from the patient, if the mConsulting service upfront payment is the same or lower than the public system, this may attract patients out of the public system. However, the ongoing care available through the public system may not be available to those using mConsulting, resulting in lower-quality or delayed care. Competition between mConsulting providers may increase the quality of their provision, at least in terms of quality of patient experience. mConsulting services could be provided by generalist, specialist or single-disease healthcare providers. Their impact will depend on how the existing health system is structured. For example, where generalist primary care is the main public healthcare provider, access to mConsulting might lead to patients going directly to specialists in the private sector, thus by-passing the public referral system and potentially incurring unnecessary costs and receiving inappropriate services. Overall, mConsulting has the potential to increase help-seeking from communities who have had poor access to healthcare – which is an advantage. However, in the context of a wider health system that has limited capacity, such a system may be unable to cope with the resulting increase in demand. This could lead to a deterioration in quality of care, with some patients with treatable conditions being turned away due to lack of resources. This is distressing for patients and can increase levels of moral distress and burn out among healthcare providers.^97,98^

Current national regulations relating to healthcare providers, and the effectiveness with which regulations are implemented, will have an impact on how mConsulting develops. National governments need to consider this, along with how to respond to requirements for cross-national provision.^18^ The potential to record consultations and review their quality is an opportunity for improving care quality, however, healthcare providers may feel threatened by this. The ease of recording mConsultations, without the other party necessarily being aware of this, is likely to have legal implications.

As health systems evolve along with mConsulting, patient-held records may provide more flexibility for patients, as they can share them with others, or transfer them to providers of their choice.

## Discussion

There is a lack of evidence on the impact of mConsulting for those living in urban slums and those in rural locations, where there is little quality healthcare provision, as most research on mConsulting has been undertaken where quality healthcare already exists. However, there is potential for mConsulting to contribute towards the urgently needed ‘revolution’ to bring about high-quality healthcare in such settings.

In their Lancet Commission, Kruk et al. propose that high-quality healthcare is underpinned by four values: it is for people, resilient, efficient and equitable.^3^ Being for people means healthcare has to be accessible: mConsulting has the potential to improve accessibility where there is (affordable) technology, both personal and infrastructural, to support it. Furthermore, Kruk et al. suggest that people should have agency over their healthcare decisions and be able to hold healthcare providers to account.^3^ This may be easier through the technology that supports mConsulting, giving people more choice over whether, when and how they seek healthcare: where there are multiple providers, people can move between them if unhappy with the quality of care; the technical capacity to record the content of mConsultations opens up the potential to expose poor quality care. Quality healthcare is person-centred, despite the asymmetry of knowledge as power between patient and provider.^3^ The availability of online health information and advice, where this can be accessed, tips the balance of power towards the patient, as does the ability to hold the provider to account. Quality healthcare requires motivated healthcare providers, operating within safe and supportive work environments.^3^ It may be easier to provide such environments for mConsulting providers as they can be remote from patients, away from difficult environments, with access to support, information and experts, and do not need to be concerned with waiting room queues. With resilient technological infrastructure, there is the potential for mConsulting healthcare provision to be resilient, reorganising to deal with challenges and crises. This requires a flexible workforce and good leadership, as for all forms of healthcare.^3^ Potentially, a remote workforce may be deployed more flexibly than a workforce committed to a particular healthcare space. mConsulting is potentially more efficient for the patient, as they may not need to travel to a health facility, consequently saving time, costs and reducing disruption to economic and other day-to-day activities; this could also prove more cost-effective for healthcare providers through saving travel time and costs. However, there is also the potential for inefficiencies and hazards and it is unclear whether mConsulting has the potential to be equitable – available and affordable for everyone, whatever their socio-economic status. Equitable, efficient mConsulting may become increasingly possible as infrastructure improves and the cost of phones and airtime decrease. However, there is also the risk of increased demand for services that are inappropriate or unavailable in the context. Furthermore, the importance of synchronous human interaction may be systematically different for different types of issues (e.g. cancer diagnosis, self-limiting viral illness). This means that the effectiveness of mConsulting will not be uniform across conditions, which has the further consequences that medical outcomes will be affected and that information about prevalence and success of advice may also be distorted.^39^ We suggest that it is in the detail of how mConsulting is deployed, what it is deployed for, and who is deploying/seeking it that will make the difference to whether mConsulting attains the values of being for people, resilient, efficient and equitable.

We suggest that provision and use of mConsulting in spatially marginalised and poor populations may stimulate movement towards the UN’s sustainable development goal 3 (SDG3)^1^ of good health and wellbeing, by providing access to quality healthcare. Furthermore, there is the potential for it to contribute to further such goals, for example, reducing inequalities in access to healthcare (SDG10); in some cultures, mConsulting may empower women to both access and provide healthcare (SDG5); sustainability of remote rural communities reducing migration to urban areas (SDG11); and movement towards strong governance and regulation (SDG 16). Its deployment, often requiring partnership between public and private sections (SDG 17), may stimulate the establishment of resilient infrastructure, promote sustainable industrialisation and foster innovation (SDG9), and stimulate economic growth and availability of decent work (SDG8). However, while mConsulting fits with the aspirations of sustainable development and the provision of high-quality person-centred health systems, this requires resources, alongside critical attention to the needs of local communities in specific settings.
